# Case Report: An extremely rare penile shrapnel injury

**DOI:** 10.3389/fped.2023.1281413

**Published:** 2024-01-08

**Authors:** Renbing Pan, Jingwen Liu, Lijun Wan, Jianyong Zhu

**Affiliations:** ^1^ Department of Urology, The Quzhou Affiliated Hospital of Wenzhou Medical University, Quzhou People’s Hospital, Quzhou, Zhejiang, China; ^2^ Department of Psychiatry, Shulan Quzhou Hospital, Quzhou, Zhejiang, China

**Keywords:** penis, shrapnel injury, erectile function, dysuria, neurovascular bundle

## Abstract

Penile shrapnel injuries are an exceedingly rare occurrence and a medical emergency. Herein, we present a case of penile shrapnel wounds in an adolescent male and discuss the management and complications associated with penetrating injuries to penile. We reported that an 18-year-old Chinese armed police soldier underwent debridement, shrapnel removal and suturing under spinal anesthesia. Six days postoperatively, he was discharged from the hospital smoothly. The patient reported normal erectile function and urination following discharge. With a follow-up of three months, the patient exhibited no symptoms of dysuria or erectile dysfunction. It is explicitly stated that prompt surgery intervention described in this report resulted in optimal prognosis. Penile shrapnel injury is a rare phenomenon typically associated with emergency drill and military training involving explosive shells. With regard to penetrating penile injury, timely surgical exploration is essential because it avoids penile plaque formation, penile fibrosis and angulation, and accelerates the return to erectile and urination function.

## Introduction

In an era of peace, occurrences of penile shrapnel injuries are extremely rare. Various mechanical penile injuries and wound complications have been extensively documented in the literature (1–4). However, there is no previous record of reported cases regarding penile shrapnel injuries in published literature. Penile shrapnel injuries can involve the corpus cavernosum and urethra. Herein, we describe a case of an 18-year-old man with a shrapnel injury to his penis. We discuss the management of such penetrating injuries in both emergency and urology departments, including the entire surgical procedure involved. Although exceptionally rare, familiarity with penetrating penis injuries can mitigate their long-term repercussions on urogenital system and sexual health (5). Given the rarity and complexity of penetrating penile injuries, we herein present a case of penile shrapnel injury along with our commentary on subsequent surgical management and patient recovery. The authors believe that this introduction of a surgical approach may enhance our experience and expertise in managing future cases involving penetrating injuries of the penis.

## Case presentation

**Figure 1:**
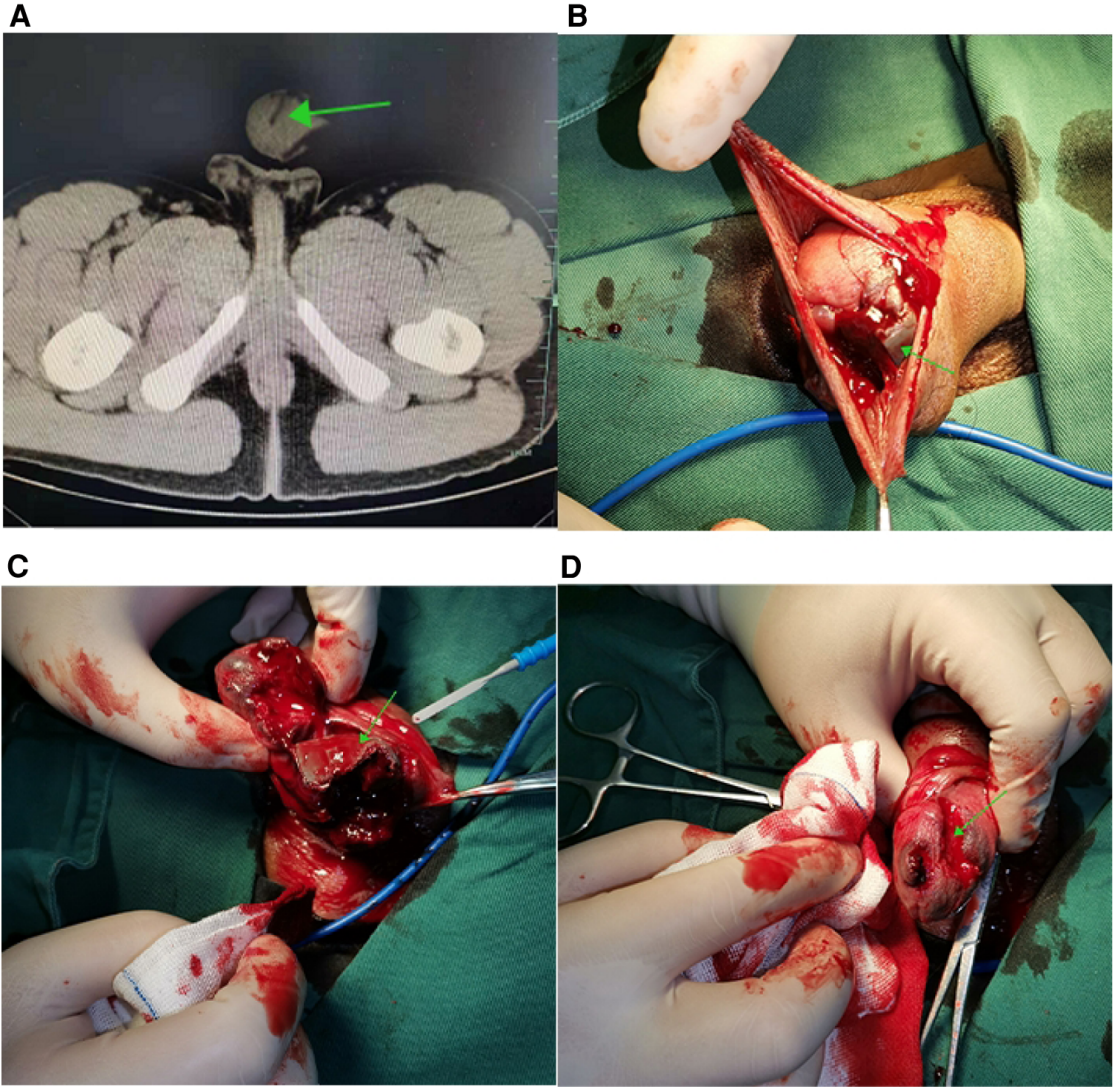
(**A**) CT scan demonstrating a foreign body penetrated the corpora cavernosum; (**B**) an irregular shrapnel penetrated from the balanus to the corpora cavernosum; (**C,D**) the shrapnel penetrated very deeply with active bleeding and removed the shrapnel carefully.

**Figure 2:**
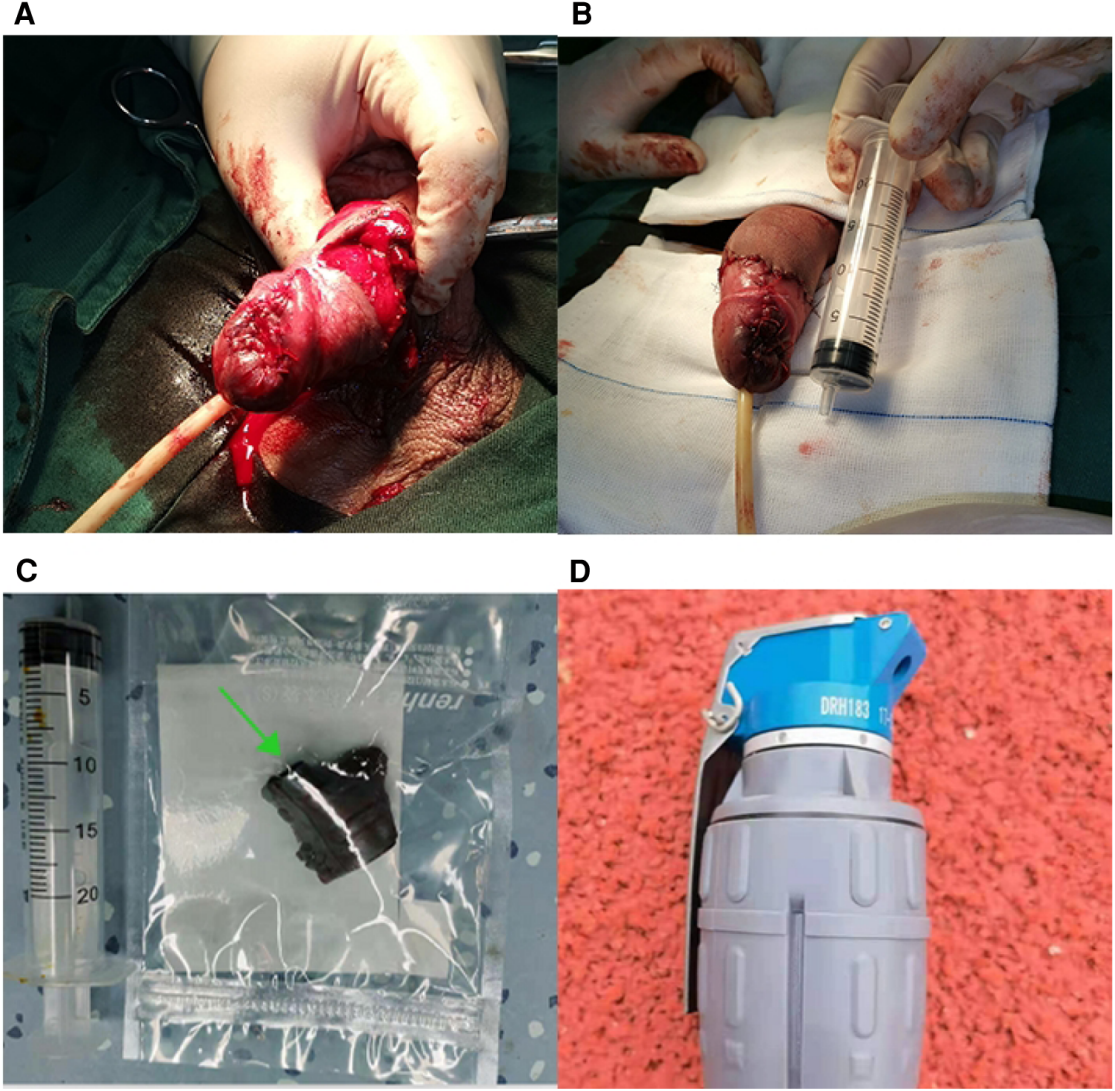
(**A,B**) Repair and reconstruction of the corpus cavernosum and circumcision; (**C**) a 1.5 × 2.0 cm ragged shrapnel covered in blood was removed intactly; (**D**) the sample of stun grenades.

An 18-year-old Chinese armed police soldier accidentally blew a piece of shrapnel into his penis while taking part in an emergency exercise using a detonation bomb. He was admitted to emergency room of our hospital immediately by his colleagues. On the admission he was alert but terrified. He felt swelling and pain in his penis. More blood oozed from his penis. There was no sign of injury to the scrotum. His vital signs were within normal limits. We did not see the shrapnel on the penile surface. But we could touch an irregular hard object by penile palpation. Coincidentally, there was no damage to any part of his body except his penis. CT scan showed a 1.5 × 2.0 cm shrapnel penetrating the corpora cavernosum, with associated tumidness and pneumatosis (Figure 1A). He was urgently transported to the operating room and underwent debridement, shrapnel removal, and suturing under spinal anesthesia. We found an irregular shrapnel penetrating from the balanus to the corpora cavernosum with active bleeding at the bottom of rift (Figures 1B,C). It was very fortunate for the patient that the shrapnel did not damage the anterior urethra. We removed the shrapnel cautiously (Figures 1D,2A–C) and repaired the cavernous body of the penis and the glans successfully. Due to the defect of the foreskin caused by blast injury, we did a circumcision incidentally (Figures 2A,B). We gave cefuroxime for 2 days to prevent potential infection from exogenous foreign material. The patient had a normal penile erection on the first postoperative day. After a period of convalescence, the patient was discharged to home on the 5th postoperative day. Upon discharge, the patient reported normal erection and demonstrated a normal urination. No sex dysfunction and urination dysfunction were observed during three months follow-up.

## Discussion and review of the literature

Shrapnel injuries are a rare damage, rarely previously reported in the chest, heart, skull, abdomen and so on (6). However, penile shrapnel injury is an extremely rare condition. So far, there have been no reported cases of penile shrapnel injuries worldwide. To our knowledge, this is the first reported of penile shrapnel penetrating injury.

Genitourinary system injuries are usually caused by penetrating and blunt trauma. The urogenital system accounts for approximately 10% of all injuries encountered in the emergency department. The male genitourinary system is effectively safeguarded against blunt and penetrating injuries by the surrounding internal organs, musculoskeletal structure, and natural mobility, in addition to its external reproductive organs (7). The management of male genital penetrating trauma is considered a urological emergency due to the substantial risk of infection and the prioritization of safeguarding endocrine, sexual, and fertility functions (8). Approximately 35% of penetrating urogenital injuries affect the external genitalia, such as penis or scrotum (9). The objective of treating these types of injuries is to protect the integrity of the urethra and sexual function (10). We herein present an armed forces soldier suffered a serious penile penetration injury accidentally due to military training with explosive shells.

The corpora, penile soft tissue or urethra can be involved in cases of penetrating penile injuries (11). Previous studies have indicated that up to 80% of penetrating penile injuries are accompanied by associated injuries, with the groin area and scrotum being the most commonly affects sites, followed by urethral injuries as the second most frequent occurrence (12). For this patient, we routinely performed catheterization. However, all of the penile penetrating injuries require concern for urethral injury, and cystoscopy or retrograde urethrography is routinely recommended. Moreover, a possible urethral trauma is a contraindication for catheterization, and this is also something that we need to pay attention to when dealing with similar cases in the future. Furthermore, due to the neurovascular bundle (NVB) starts at the lateral angle of the seminal vesicle, passes posterolateral of the prostate gland, and ends at the cavernous body of the penis (13), we also need to be concerned about the possibility of damage to the penile neurovascular bundle. Fortunately, this patient had normal erectile function after surgery. In addition, abdominal imaging should be performed as part of a complete trauma assessment in all patients with mechanical foreign body injuries where no associated primary injury is suspected (11). To this patient, an abdominal CT was performed immediately and successful indwelling catheterization was accomplished at the emergency room. Fortunately, there was no damage to any part of his body except his penis, and no significant urethral injury was found during the operation.

In this case report, we describe an unprecedented penis shrapnel injury. The significance of this case is that an isolated piece of shrapnel had lodged in the soldier's penis, causing the distal corpus cavernosum to rupture. Literature recommend early surgical exploration of penis can alleviate pain and reduce the risk of erectile dysfunction, corpora cavernosa fibrosis, and penile symptomatic scarring or recurrence (14). Through our timely surgery to remove the shrapnel completely, the patient had a normal penile erection on the first postoperative day. During the follow-up of up to three months, the patient had no urinary abnormalities or erectile dysfunction and healed well.

Due to the strict supervision, stun grenades (Figure 2D) are barely visible in Chinese market and are commonly used by the military and police. It is commonly used by police or special force for rescue, raid and so on. The detonator, which explodes with a loud sound, is used to deter lawbreakers, temporarily rendering them deaf and disoriented, and thus incapable of resistance. Under normal circumstances, it does not form shrapnel or shock waves, so it will not bring permanent damage to the surrounding personnel. Unfortunately, on account of an accidental impact during the dropping of the stun grenades, this patient was inadvertently hit in penis by shrapnel through his trousers. In the future, we should strength the supervision of ammunition weapon, ensure the safe and standardized use, and eliminate potential security hazard.

## Conclusion

We report a case of penetrating penile injury with an irregular shrapnel. After shrapnel removal, the solider performed well and no acute complications were found on in-hospital examination. Once penile injury occurs, it should be properly diagnosed and treated. Surgical exploration is recommended because it avoids penile plaque formation, penile fibrosis, and angulation, and accelerates the return to normal penile function. Furthermore, surgeons should be aware of the complications of penetrating penile injuries, as missing an injury can have significant long-term consequences for genitourinary and sexual health. Therefore, prompt urological consultation and regular follow-up are required in almost all of these cases.

## Data Availability

The original contributions presented in the study are included in the article/Supplementary Material, further inquiries can be directed to the corresponding authors.
